# Wnt inhibition promotes vascular specification of embryonic cardiac progenitors

**DOI:** 10.1242/dev.159905

**Published:** 2018-01-01

**Authors:** David E. Reichman, Laura Park, Limor Man, David Redmond, Kenny Chao, Richard P. Harvey, Makoto M. Taketo, Zev Rosenwaks, Daylon James

**Affiliations:** 1 Center for Reproductive Medicine, Weill Cornell Medical College, New York, NY 10065, USA; 2 Institute for Computational Biomedicine, Weill Cornell Medical College, New York, NY 10065, USA; 3 Developmental and Stem Cell Biology Laboratory, Victor Chang Cardiac Research Institute, Darlinghurst, NSW 2010, Australia; 4 St. Vincent's Clinical School, University of New South Wales, Kensington 2052, Australia; 5 School of Biological and Biomolecular Sciences, University of New South Wales, Kensington 2052, Australia; 6 Department of Pharmacology, Graduate School of Medicine, Kyoto University, Sakyo, Kyoto 606-8501, Japan; 7 Tri-Institutional Stem Cell Derivation Laboratory, Weill Cornell Medical College, New York, NY 10065, USA

**Keywords:** Cardiac development, Cardiac differentiation, Wound healing, Myocardial infarction, Myocardial scar formation, Endothelial differentiation, Human embryonic stem cells, Cardiovascular progenitor, Wnt signaling, Non-canonical Wnt, Wnt5a, Mouse

## Abstract

Several studies have demonstrated a multiphasic role for Wnt signaling during embryonic cardiogenesis and developed protocols that enrich for cardiac derivatives during *in vitro* differentiation of human pluripotent stem cells (hPSCs). However, few studies have investigated the role of Wnt signaling in the specification of cardiac progenitor cells (CPCs) toward downstream fates. Using transgenic mice and hPSCs, we tracked endothelial cells (ECs) that originated from CPCs expressing NKX2.5. Analysis of EC-fated CPCs at discrete phenotypic milestones during hPSC differentiation identified reduced Wnt activity as a hallmark of EC specification, and the enforced activation or inhibition of Wnt reduced or increased, respectively, the degree of vascular commitment within the CPC population during both hPSC differentiation and mouse embryogenesis. Wnt5a, which has been shown to exert an inhibitory influence on Wnt signaling during cardiac development, was dynamically expressed during vascular commitment of hPSC-derived CPCs, and ectopic Wnt5a promoted vascular specification of hPSC-derived and mouse embryonic CPCs.

## Abstract (precis)

**Summary:** Non-canonical inhibitory Wnt signaling via Wnt5a fosters endothelial differentiation of Nkx2.5^+^ cardiac progenitor cells during mouse embryogenesis and human pluripotent stem cell differentiation.

## INTRODUCTION

One out of every three deaths in the USA results from cardiovascular disease (Roger et al., 2012). Despite the epidemic scale of predisposing conditions (hypertension, smoking, obesity), major advances in the surgical treatment of cardiovascular disease have plateaued. Regenerative therapies represent a promising alternative to surgical and/or pharmacological interventions for cardiovascular disease, but wound healing mechanisms unique to the heart undermine the restorative contribution of endogenous or exogenous cells (Cleutjens et al., 1999). Autologous, tissue-resident cardiac progenitor cells (CPCs) have demonstrated modest benefit upon transplantation into patients following myocardial infarction (MI) (Bolli et al., 2011; Chugh et al., 2012), and cardiomyocytes differentiated from human pluripotent stem cells (hPSCs) and transplanted into small animal models of MI have shown favorable results (Laflamme et al., 2007; Shiba et al., 2012). However, non-fatal ventricular arrhythmias have been noted in non-human primates engrafted with hPSC-derived cardiomyocytes (Chong et al., 2014), underscoring the challenge of achieving ordered intercalation of exogenous cells into injured cardiac tissue.

Redirection of cardiac wound healing processes in adult hearts might be a favorable approach for improving cardiac function following ischemic injury. Adult zebrafish and neonatal mice are capable of undergoing constructive heart regeneration (Uygur and Lee, 2016), and recapitulation of these processes to redirect cell fate could promote more constructive, pro-angiogenic wound healing. Indeed, recent work has demonstrated the potential of this approach in mouse models of cardiac ischemia (Zangi et al., 2013), and other studies have identified novel mediators of cardiac regenerative processes that might be exploited in injured heart. Yet, relatively little is known of the signaling pathways that drive vascular fate within the CPC population in embryogenesis or adulthood.

Among the signal transduction pathways that play a prominent role in cardiac development and differentiation, the Wnt pathway has been extensively studied. Since early experiments in chick (Marvin et al., 2001) and frog (Schneider and Mercola, 2001) showed that inhibition of Wnt is necessary for induction of cardiogenesis, many groups have confirmed that naturally occurring and synthetic Wnt inhibitors can drive the differentiation of NKX2.5^+^ populations in mouse embryos and pluripotent stem cell cultures (Elliott et al., 2011; Lian et al., 2012; Naito et al., 2006; Paige et al., 2010; Qyang et al., 2007; Willems et al., 2011). Collectively, these studies have demonstrated a multiphasic role for Wnt signaling in cardiac fate choice determination (Gessert and Kuhl, 2010): activation of Wnt is required during gastrulation for induction of cardiac mesoderm, while inhibition of Wnt signaling later enables differentiation of cardiac progenitors. Recent studies have demonstrated a further role for canonical Wnt signaling in the specification of epicardial progenitor cells from NKX2.5^+^ precursors (Iyer et al., 2016; Witty et al., 2014); however, few studies have specifically addressed the role of Wnt signaling in the designation of endothelial cell (EC) fate; GSK3β inhibitors have been used in chemically defined medium to robustly induce differentiation of CD34^+^ CD31 (PECAM1)^+^ endothelial progenitors from hPSCs (Bao et al., 2015; Lian et al., 2014), and a high-content screening assay identified numerous pharmacological inhibitors of Wnt that promoted the differentiation of cardiomyocytes, and no other mesodermal derivative, from hPSCs (Willems et al., 2011). Yet these studies incorporated Wnt inhibition into heterogeneous cultures of differentiating hPSCs during a relatively early and/or broad temporal window. As such, the function of Wnt specifically in the designation of cardiac EC, myocardial, smooth muscle or fibroblast fate from multipotent CPCs remains unclear.

Here, we have generated a dual reporting hPSC line that identifies NKX2.5^+^ (cardiac) and VE-cadherin (CDH5)^+^ (endothelial) derivatives during differentiation. Utilizing this line and a cross of vascular [*Flk1-GFP* (Ema et al., 2006)] and cardiac [*Nkx2.5*^Cre^ (Stanley et al., 2002)] mouse reporter strains, we specifically identified and tracked ECs that originated from cardiac progenitors *in vitro* and *in vivo*. Expression analysis and live monitoring of Wnt activity during hPSC differentiation and *in vivo* cardiogenesis revealed a correlation between reduced Wnt activity and acquisition of EC identity, and inhibition of Wnt signaling promoted vascular specification of hPSC-derived and mouse embryonic CPCs. Finally, gain-of-function experiments in hPSC cultures and mouse embryos revealed a function for WNT5A, the non-canonical Wnt effector, in the vascular specification of CPCs. These data elucidate a novel influence on EC specification from cardiac-specific progenitors and identify Wnt signal inhibition via WNT5A as a potential driver of neovascularization in the developing heart.

## RESULTS

### Demarcation of vascular commitment from NKX2.5-expressing hPSC derivatives

**Fig. 1.**
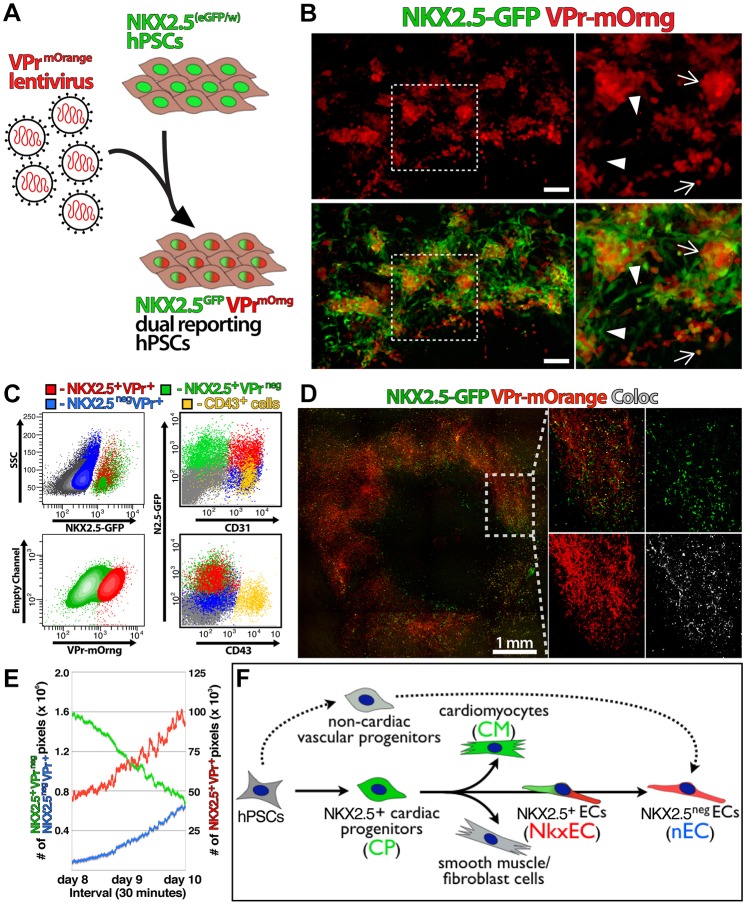
**Demarcation of vascular commitment** **from NKX2.5-expressing hPSC derivatives.** (A) The *NKX2.5*^eGFP/w^ hPSC line was targeted with lentiviral particles encoding VPr^mOrange^, an EC-specific promoter sequence driving mOrange fluorescent protein. (B) Following 10 days of serum-free differentiation, a colony of hPSC derivatives was scanned by confocal microscopy. Boxed regions are shown at higher magnification to the right. Arrows indicate endothelial cells that are also GFP positive. Arrowheads indicate cells that are single positive for the NKX2.5-GFP reporter. (C-E) Live images of differentiation cultures were captured in sequential time-lapse culture from days 8 to 10; representative populations isolated from differentiation at day 10 are shown (D) and quantification of NKX2.5^+^ VPr^neg^ (green), NKX2.5^neg^ VPr^+^ (blue) and NKX2.5^+^ VPr^+^ (red) pixel density is shown (E). Coloc, colocalization. (F) Dual reporting hPSCs enable identification of unique populations that represent successive stages in the developmental hierarchy of cardiac endothelial specification. Scale bars: B, 100 µm; D, 1 mm.

To enable live tracking and longitudinal analysis of cardiac and endothelial fate acquisition in an experimentally tractable *in vitro* model, we applied an EC-specific transgenic labeling strategy based on the *CDH5* promoter [VPr (James et al., 2010)] to the cardiac-specific hPSC line *NKX2.5*^eGFP/w^ (Elliott et al., 2011) (Fig. 1A). Differentiation of this line in conditions that promote cardiovascular cell fate (Lian et al., 2012; Paige et al., 2010; Willems et al., 2011) resulted in regions in which both reporters were enriched (Fig. 1B), and flow cytometry corroborated the presence of NKX2.5^GFP+^ ECs. Indeed, live tracking of hPSC differentiation over 48 h from day 8 to 10 revealed a progressive decrease in single-positive NKX2.5^GFP+^ VPr^neg^ cells as the double-positive NKX2.5^GFP+^ VPr^mOrange+^ population increased (Fig. 1C-E, Movies 1,2). This approach enabled the live tracking and analysis of three unique populations via microscopy and flow cytometry: NKX2.5^+^ VPr^neg^ CD31^neg^ cardiac progenitors/cardiomyocytes (CP/CM, green); NKX2.5^+^ VPr^+^ CD31^+^ ECs (NkxEC, red); and NKX2.5^neg^ VPr^+^ CD31^+^ ECs that no longer express NKX2.5 or never did (nECs, blue) (Fig. 1F).

### Nkx2.5-expressing progenitors contribute significantly to endocardial ECs

**Fig. 2.**
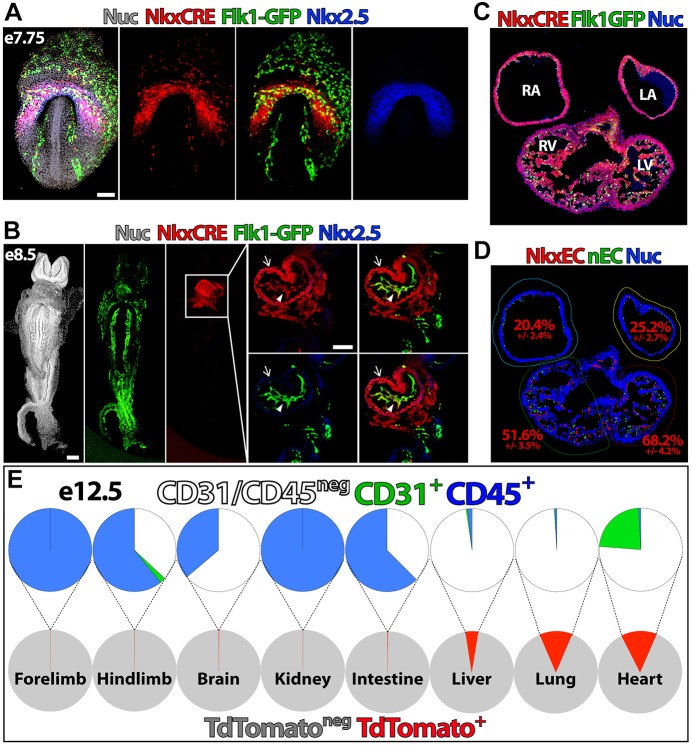
**Nkx2.5-expressing progenitors contribute significantly to endocardial ECs.** (A,B) *Flk1-GFP;Nkx2.5*^Cre^*;Rosa*^tdTom^ mouse embryos were isolated at E7.75 (A) and E8.5 (B), enabling specific identification of cells derived from Nkx2.5-expressing progenitors (red) and ECs (green). In B, arrows indicate myocardium and arrowheads indicate endocardium. (C,D) Cryosections of E11.5 heart with colocalization masking enabling quantification of the NkxEC:nEC ratio. (E) Organs isolated from *Flk1-GFP;Nkx2.5*^Cre^*;Rosa*^tdTom^ embryos at E12.5 were dissociated and the distribution of NkxECs (green) and CD45^+^ cells (blue) was quantified within the tdTomato^+^ population (red). LA, left atrium; LV, left ventricle; RA, right atrium; RV, right ventricle; Nuc, nuclei staining. Scale bars: 100 µm.

To establish a complementary lineage-tracing strategy in the mouse that specifically distinguishes ECs that arise from Nkx2.5-expressing (NkxEC) versus non-Nkx2.5-expressing (nEC) compartments, we crossed *Flk1-GFP* mice (Ema et al., 2006) with *Rosa*^tdTom^ (Madisen et al., 2010) and *Nkx2.5*^Cre^ (Stanley et al., 2002) strains. NkxECs were observed in the cardiac crescent (Fig. 2A) and endocardium (Fig. 2B), and made up a large proportion of endocardial ECs in sections of embryonic day (E) 11.5 heart (Fig. 2C), with colocalization masking (spatial overlap of cardiac and vascular-specific reporter signals) in respective cardiac chambers revealing an increased ratio of NkxECs in ventricles (Fig. 2D). To determine whether NkxECs are present in other organs during development, we isolated organs at E12.5 and enzymatically dissociated them into single-cell suspensions. Flow cytometry revealed a substantial population of tdTom^+^ cells in liver and lung; however, the NkxEC population was exclusively observed in the heart (Fig. 2E).

### RNA-sequencing (RNA-seq) of NkxECs reveals reduced Wnt activation and a tip cell phenotype

**Fig. 3.**
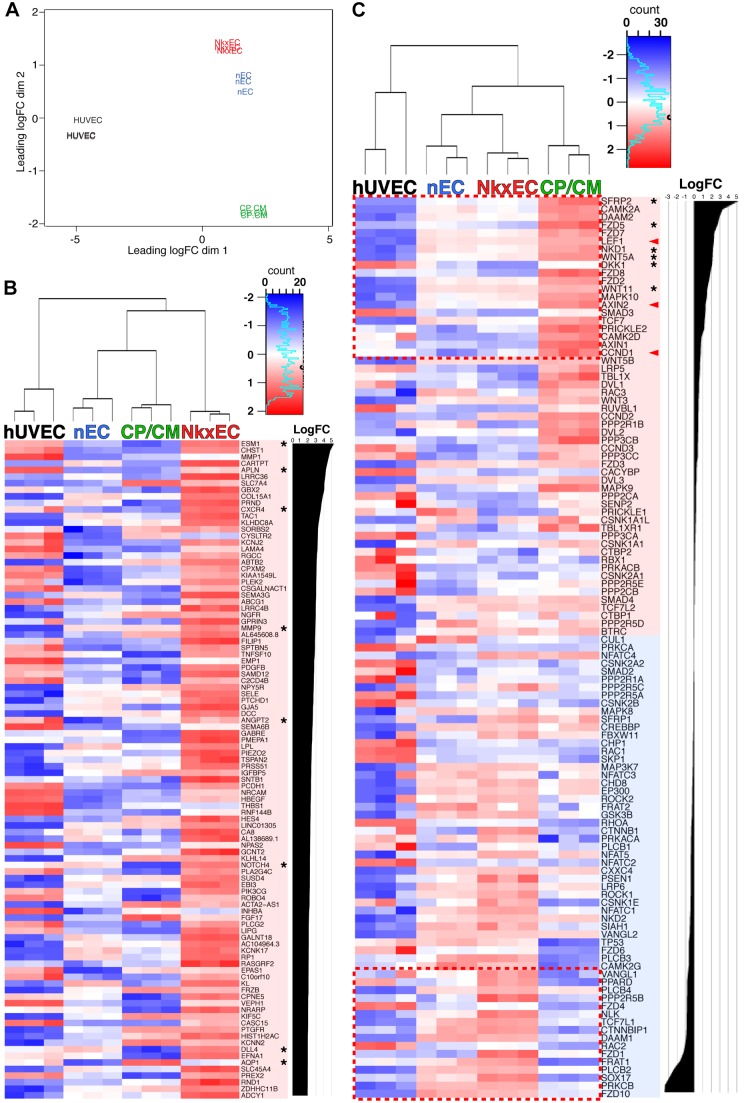
**RNA-seq of NkxECs reveals reduced Wnt activation and tip cell phenotype.** (A) RNA-seq was performed on biological triplicate samples of HUVECs, CP/CMs, NkxECs and nECs; an expression MDS plot showing clear differentiation between the four cell types is shown. (B) Heatmap showing the top 100 genes that were upregulated in NkxECs relative to nECs. (C) Heatmap showing a KEGG list of Wnt pathway components with transcripts listed according to log fold change (FC) of CP/CMs over NkxECs. Asterisks (B) specify genes known to be highly expressed in endothelial tip cells or (C) specify Wnt effectors known to play an inhibitory function. Arrowheads (C) specify canonical Wnt target genes. *P*<1×10^−5^ for all transcripts in B; the dashed boxes (C) shows transcripts that are significantly increased (upper) or decreased (lower) in CP/CMs relative to NkxECs (*P*<0.05).

Resolution of hPSC derivatives precisely at the interface of CPC and NkxEC fate enables identification of candidate signaling pathways that might play an inductive role in vascular specification within the heart. To define molecular hallmarks that are unique to NkxECs and identify signaling pathways that play a role in the specification of endocardial fate, we compared RNA-seq profiles of human umbilical vein ECs (HUVECs) with that of CP/CMs, NkxECs and nECs at day 7 of differentiation (Fig. 3). Three biological replicates of each population were isolated by FACS for RNA isolation and sequencing and an expression MDS plot revealed clear differentiation between all four populations (Fig. 3A), with direct comparison of NkxECs with nECs revealing the unique character of NkxECs. Among the top 100 genes upregulated in NkxECs (Fig. 3B) were numerous genes that have been linked to Notch signaling/tip cell phenotype (del Toro et al., 2010). Additionally, among transcripts known to be related to Wnt signaling (KEGG database), CP/CMs were highly enriched for targets of canonical Wnt (arrowheads, Fig. 3C) as well as canonical and non-canonical inhibitors (asterisks, Fig. 3C), all of which were significantly reduced upon acquisition of NkxEC fate.

### Enforced Wnt inhibition promotes NkxEC fate from hPSC-derived CPCs

**Fig. 4.**
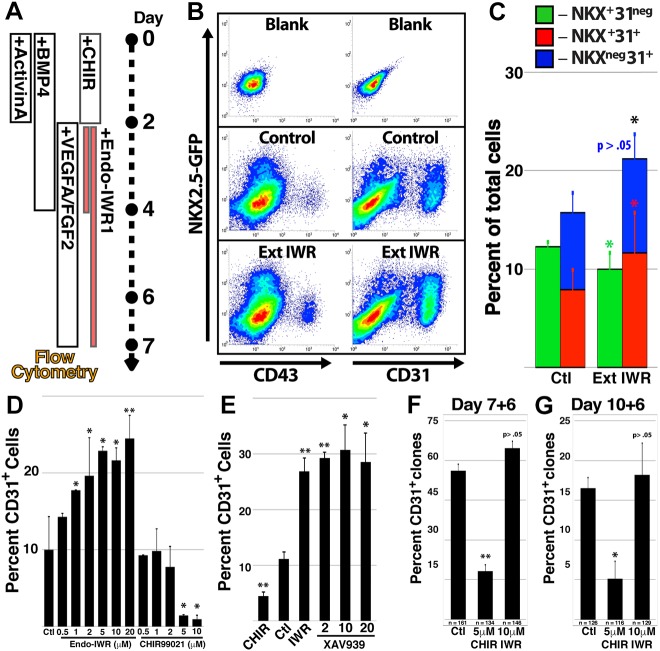
**Enforced Wnt inhibition promotes NkxEC fate from hPSC-derived CPCs.** (A-C) hPSCs were sequentially stimulated with factors that promote cardiovascular differentiation with the Wnt inhibitor Endo-IWR1 included for 2 days (days 2-4) or for an extended period (days 2-7). Colored contour plots showing representative flow cytometry measurements of the two conditions at day 7 (B) and quantification of the CP/CM, NkxEC and nEC populations (C). (D) CP/CMs were isolated at day 7 of differentiation and cultured for an additional 5 days in different concentrations of Endo-IWR1 or CHIR99021. The percentage of CD31^+^ cells in resultant cultures was determined by flow cytometry. (E) Day 7 CP/CMs were isolated and cultured in increasing concentrations of the Wnt inhibitor XAV-939. The percentage of CD31^+^ cells was quantified after 5 days. (F,G) CP/CMs were isolated at day 7 (F) or 10 (G) of differentiation and plated at clonal density. Clones exhibiting the presence of EC derivatives after 6 days were quantified in response to control, CHIR99021 or Endo-IWR1 conditions. Error bars represent s.d. between eight (C) or six (D,E) biological replicates or (F,G) between three groups of clones with the aggregate number of clones for all three groups shown beneath the bar. *P<0.05, **P<0.01, relative to control conditions.

To address the role of Wnt during vascular commitment of hPSC-derived CPCs, we augmented published protocols for cardiovascular differentiation by extending 2 days of Wnt inhibition to 5 days (Fig. 4A). Relative to control conditions, prolonged Wnt inhibition promoted an increased proportion of NkxECs and total ECs at the expense of CP/CMs (Fig. 4B,C). To isolate the effect of Wnt signal inhibition on the NKX2.5-expressing progenitor population, we purified CP/CMs by FACS at day 7 and exposed them to pharmacological modulators of Wnt signaling in escalating concentrations (Fig. 4D). Inhibition (Endo-IWR1) or activation (CHIR99021) of Wnt signaling increased or decreased, respectively, the percentage of ECs within the CP/CM-derived population. An alternative inhibitor of Wnt signaling, XAV-939, also increased the percentage of ECs arising from isolated CP/CMs (Fig. 4E).

The gold standard for demonstration of multipotency and lineage selection in cell culture is isolation and tracking of single-cell clones. To test the effect of Wnt signal inhibition at a clonal level, we distributed CP/CMs at serial dilution into culture conditions in which Wnt signaling was activated or inhibited (Fig. 4F,G). FACS deposition (see Materials and Methods) resulted in clonal outgrowths from 10.2% and 8.6% of cells isolated from day 7 (Fig. 4F) and 10 (Fig. 4G) of differentiation, respectively, and these cells were split into three groups that were cultured in medium containing VEGFA alone or combined with CHIR99021 or Endo-IWR1. After 6 days, clonal outgrowths were assessed for EC content. The percentage of clones containing ECs was significantly lower for CP/CMs isolated at day 10, perhaps reflecting a reduced volume of CPCs within the CP/CM population at later stages. Importantly, the percentage of clones that contained ECs was lower following Wnt activation at either time point, and although the percentage of EC-containing clones was only slightly increased following Wnt inhibition, it is possible that plating cells at clonal density effectively eliminates the paracrine signaling impetus present in multicellular culture, resulting in de facto Wnt inhibition under these conditions.

### Cardiac-specific gain/loss of Wnt function modulates vascular fate *in vivo*

**Fig. 5.**
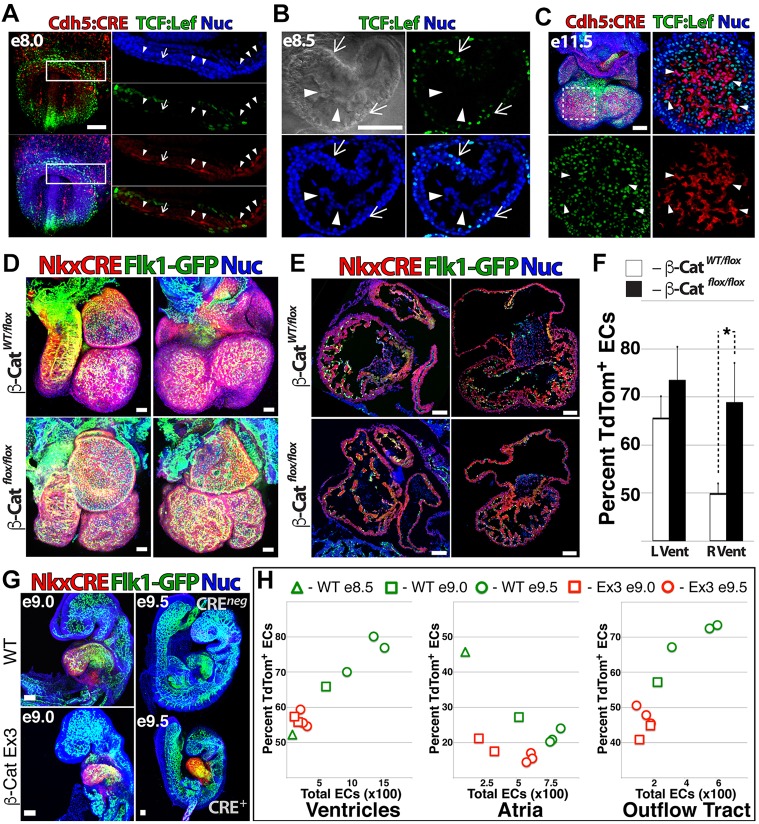
**Cardiac-specific gain/loss of Wnt function modulates vascular fate *in vivo*.** (A-C) Whole-mount confocal images of *TCF/Lef:H2B-GFP;Cdh5*^Cre^*;Rosa*^tdTom^ mouse embryos at E8.0 (A) and E8.5 (B) and isolated heart at E11.5 (C). Arrowheads (A-C) indicate ECs; arrows indicate (A) an EC that is faintly positive for *TCF/Lef:H2B-GFP* or (B,C) *TCF/Lef:H2B-GFP*^+^ cells. (D,E) The β-catenin^LOF^ strain was crossed with *Flk1-GFP;Nkx2.5*^Cre^*;Rosa*^tdTom^ mice to generate embryos with heterozygous and homozygous ablation of β-catenin. *z*-stack projections are compared (D) and sagittal and coronal sections are shown (E). (F) The percentage of NkxECs was quantified for the left and right ventricles of β-catenin^LOF^ versus heterozygous littermates. Error bars represent s.d. between five biological replicates. *P<0.05. (G,H) β-catenin^GOF^ males were crossed with *Flk1-GFP;Nkx2.5*^Cre^*;Rosa*^tdTom^ females to generate embryos with constitutively active Wnt signaling in the heart. β-catenin^GOF^ embryos versus control littermates are shown at E9.0 and E9.5 (G) and total EC number and percentage NkxECs were measured for ventricles, atria and outflow tract of β-catenin^GOF^ versus control littermate hearts between E8.5 and E9.5 (H). Scale bars: A-E,G, 100 µm.

To determine whether Wnt signaling is linked to cardiac EC specification *in vivo*, we crossed *TCF/Lef:H2B-GFP* mice (Ferrer-Vaquer et al., 2010), which provide single-cell resolution of Wnt signaling status, with a strain carrying an EC-specific Cre recombinase [*VEC-Cre* (Chen et al., 2009), referred to here as *Cdh5*^Cre^] and *Rosa*^tdTom^ (Madisen et al., 2010). Within the cardiac crescent of these embryos at ∼E8.0 (Fig. 5A), Wnt was variably activated; however, nascent ECs were uniformly negative for H2B-GFP, apart from rare cells (arrow, Fig. 5A) in which H2B-GFP was faintly observed. Wnt activation and EC identity were also mutually exclusive upon establishment of endocardial ECs at E8.5 (Fig. 5B) and later in ECs within the ventricles of the E11.5 heart (Fig. 5C).

To measure the effect of cardiac-specific gain or loss of Wnt function on the NkxEC:nEC ratio, we crossed *Flk1-GFP;Nkx2.5*^Cre^*;Rosa*^tdTom^ mice with *Ctnnb1*^Exon3^ mice (Harada et al., 1999) (β-catenin^GOF^) and *Ctnnb1*^flox/flox^ mice (Brault et al., 2001) (β-catenin^LOF^). At E11.5, β-catenin^LOF^ embryos were grossly normal, but showed malformed atria, reduced ventricular cell density and septal defects (Fig. 5D,E, Movie 3). Quantification of EC volume in cryosections revealed an increased percentage of NkxECs in the right ventricle (Fig. 5F). β-catenin^GOF^ embryos were grossly aberrant at E11.5 (Movie 4) and clear differences relative to control littermates began to manifest at E9.0, with affected embryos exhibiting smaller size, defective heart looping and reduced vascular density within the heart (Fig. 5G, Movies 5,6). In contrast to the increased percentage of NkxECs present in β-catenin^LOF^ hearts, β-catenin^GOF^ hearts showed the reverse trend beginning at E9.0, with a significantly reduced total EC number and proportion of NkxECs in ventricles, atria and outflow tract of looping hearts (Fig. 5H).

### Non-canonical Wnts promote vascular specification of hPSC-derived CP/CMs

**Fig. 6.**
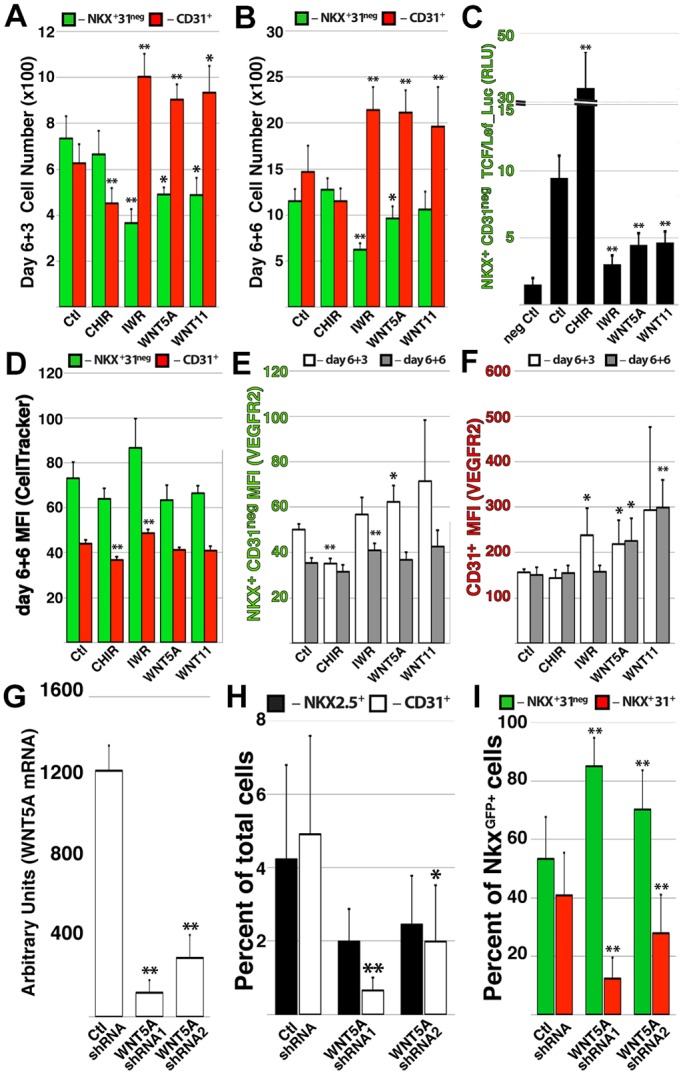
**Non-canonical Wnt signaling promotes vascular specification of hPSC-derived CPCs.** (A,B) The CP/CM population was isolated at day 6 and cultured in the presence of CHIR99021, Endo-IWR1 or recombinant WNT5A or WNT11; the total number of NKX2.5^GFP+^ CD31^neg^ and CD31^+^ cells was quantified after 3 (A) and 6 (B) days. (C) The CP/CM population was isolated at day 6 of differentiation and transduced with lentiviral particles containing a luciferase reporter driven by a *TCF/Lef*-based promoter; the activity of the reporter within NKX2.5^GFP+^ CD31^neg^ derivatives was measured after 4 days. (D-F) CP/CMs were isolated at day 6 of differentiation and incubated with CellTracker reagent to monitor cell proliferation; after 6 days in various growth conditions, the mean fluorescent intensity of the CellTracker reagent was measured among NKX2.5^GFP+^ CD31^neg^ and CD31^+^ derivatives. (E,F) Following 3 or 6 days of CP/CM culture in various conditions, the mean fluorescent intensity of FLK1 was measured on NKX2.5^GFP+^ CD31^neg^ (E) and CD31^+^ (F) derivatives. (G-I) Endogenous *WNT5A* transcript level was reduced in hPSCs using lentiviral shRNA (G), resulting in a reduced percentage of ECs among hPSC derivatives (H) and a reduced proportion of ECs within the NKX2.5^GFP+^ population (I). Error bars represent s.d. between six (A-F) or five (G-I) biological replicates. *P<0.05, **P<0.01.

In search of physiological influences that govern vascular specification within the heart, we examined global transcriptional analysis of NkxECs. In addition to downstream targets of Wnt activation, the two major ligands that mediate a non-canonical inhibitory influence on Wnt signaling, WNT5A and WNT11, were enriched in CP/CMs (Fig. 3C). Based on these data we hypothesized that non-canonical paracrine signaling elaborated by the expanding pool of CP/CMs might function to inhibit Wnt activation, thereby balancing proliferation with vascular specification. To test this, we FACS isolated CP/CMs at day 6 and cultured them in the presence of recombinant WNT5A and WNT11. Similarly to Endo-IWR1, CP/CMs cultured with WNT5A and WNT11 generated significantly increased CD31^+^ ECs at the expense of NKX2.5^+^ CD31^neg^ derivatives at 3 and 6 days post isolation (Fig. 6A,B).

Supporting the inhibitory effect of these non-canonical ligands on Wnt activation status, CP/CMs transduced with a lentiviral reporter based on the Wnt-responsive *TCF/Lef* promoter element exhibited a decrease in activity in the presence of WNT5A and WNT11 that was comparable to that caused by Endo-IWR1 (Fig. 6C). Expansion of CP/CM-derived ECs in the presence of WNT5A and Wnt11 did not occur via proliferative expansion, as CellTracker reagent introduced into CP/CMs upon their isolation was retained at levels equal to that of the control (Fig. 6D). However, surface expression of FLK1 was increased in response to WNT5A, resulting in increased mean fluorescence intensity of signal in resultant ECs (Fig. 6E,F). Finally, knockdown of endogenous *WNT5A* via lentiviral shRNA during hPSC differentiation (Fig. 6G) decreased the percentage of total ECs among differentiated derivatives (Fig. 6H), while increasing the yield of CP/CMs at the expense of NkxECs within the NKX2.5^GFP+^ population (Fig. 6I).

### Wnt5a gain of function enhances vascular specification of Nkx2.5-expressing CPCs

**Fig. 7.**
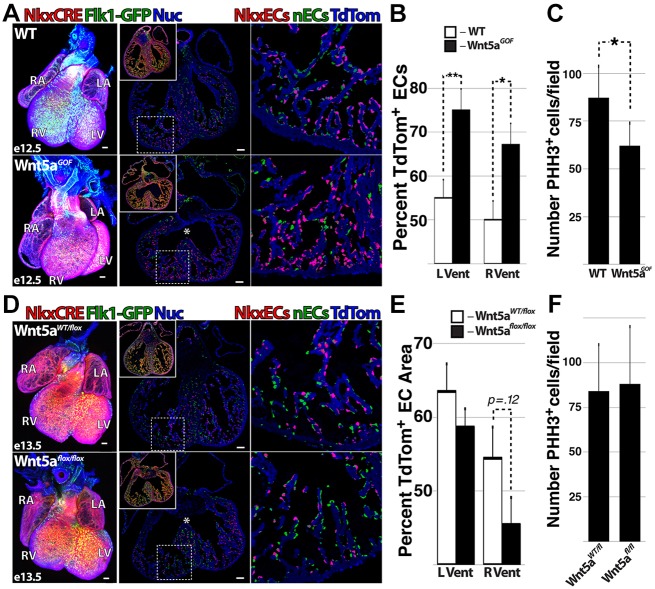
**Wnt5a gain of function enhances vascular specification of Nkx2.5-expressing CPCs.** (A-C) *Flk1-GFP;Nkx2.5*^Cre^*;Rosa*^tdTom^ mice were crossed with the *Wnt5a*^GOF^ strain. Whole-mount and coronal views of control and *Wnt5a*^GOF^ hearts at E12.5 (A), and the percentage of NkxECs (B) and frequency of phospho-histone H3-positive cells per field (C) were quantified in control and *Wnt5a*^GOF^ hearts. (D-F) *Flk1-GFP;Nkx2.5*^Cre^*;Rosa*^tdTom^ mice were crossed with the *Wnt5a*^flox/flox^ strain. Whole-mount and coronal views of *Wnt5a*^WT/flox^ and *Wnt5a*^flox/flox^ hearts at E13.5 (D), and the percentage of NkxECs (E) and frequency of phospho-histone H3-positive cells per field (F) were quantified in *Wnt5a*^WT/flox^ and *Wnt5a*^flox/flox^ hearts. Error bars represent s.d. between five biological replicates; in C,F counting 1 out of every 12 sections (10 µm thickness). Colocalization threshold masks denoting NkxECs (red) and nECs (green) are shown (A,D), with merged fluorescence images shown in the inset. Regions within dashed boxes (A,D) are magnified to the right. Asterisks indicate septal defects in mutant mice. Scale bars: 100 µm.

To elaborate on *in vivo* gain- and loss-of-function experiments (Fig. 5) and assess the role of non-canonical Wnt signaling in directing vascular fate of CPCs, we crossed *Flk1-GFP;Nkx2.5*^Cre^*;Rosa*^tdTom^ mice with the *Igs1^tm6(CAG-Bgeo,-Wnt5a)Nat^* (*Wnt5a*^GOF^; see Materials and Methods) and *Wnt5a*^flox/flox^ (Ryu et al., 2013) strains, enabling constitutive expression or deletion of *Wnt5a* in Nkx2.5-expressing cells and their derivatives (Fig. 7). Live-born pups containing *Nkx2.5*^Cre^ and *Wnt5a*^GOF^ or *Wnt5a*^flox/flox^ alleles were not observed (data not shown). Examination of E12.5 *Flk1-GFP;Nkx2.5*^Cre^*;Rosa*^tdTom^*;Wnt5a*^GOF^ embryos revealed malformed atria, reduced ventricular cell density and septal defects (Fig. 7A, Movie 7). Quantification of ECs via colocalization masking in the ventricles of mutant versus wild-type littermates revealed an increased ratio of NkxECs in *Wnt5a*^GOF^ hearts (Fig. 7B), whereas the incidence of mitotic cells in *Wnt5a*^GOF^ hearts was reduced (Fig. 7C). Notably, *Flk1-GFP;Nkx2.5*^Cre^*;Rosa*^tdTom^*;Wnt5a*^flox/flox^ analyzed at E13.5 also exhibited septal defects and aberrant development of the right ventricle (Fig. 7D), but the relative enrichment of NkxECs observed in the context of *Wnt5a*^GOF^ was reversed (Fig. 7E). However, the reduction in NkxECs in ventricles of *Wnt5a*^flox/flox^ hearts was not significant and proliferation was not affected (Fig. 7F), suggesting potential compensation for loss of Wnt5a by the closely related Wnt11.

## DISCUSSION

**Fig. 8.**
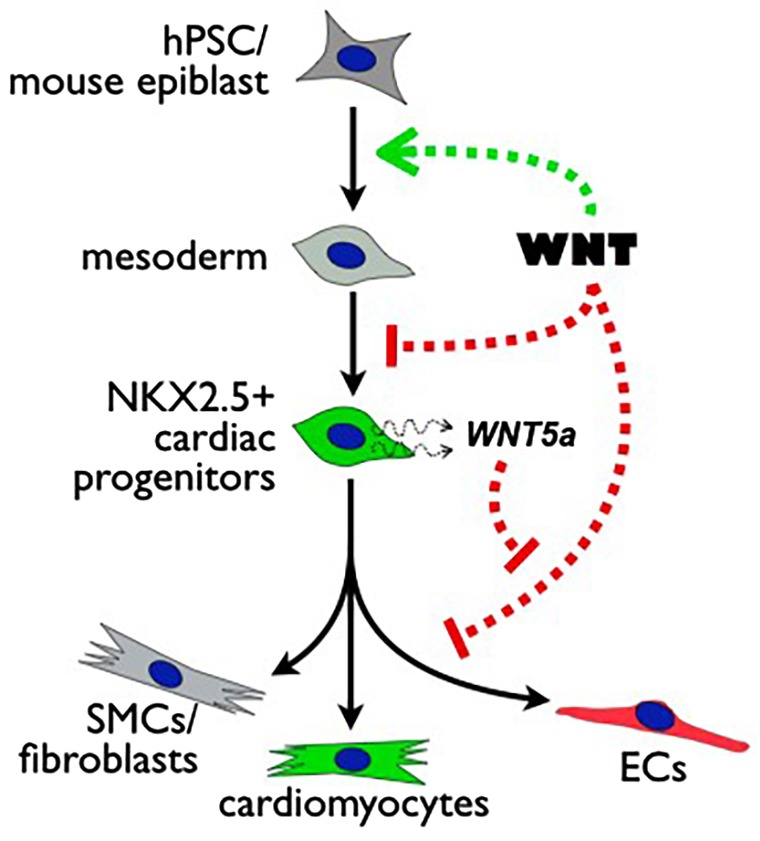
**Multiphasic function of intersection of canonical and non-canonical Wnt signaling during cardiovascular lineage diversification.** Wnt plays multiple roles during differentiation of pluripotent cells within the cardiovascular lineage. Wnt signaling initially directs pluripotent cells toward cardiac mesoderm, but is later inhibited during specification of cardiac progenitor cells expressing Nkx2.5. Subsequently, inhibition of Wnt signaling within the Nkx2.5^+^ pool via non-canonical Wnt5a promotes vascular specification.

Owing to the multiphasic influence of the Wnt pathway during embryogenesis, as well as the diversity of signaling modulators that function during development, clearly defining the role of Wnt at each milestone of cardiogenesis presents a major challenge. Here, we interrogated the role of the Wnt pathway in specifying endothelial derivatives of embryonic CPCs and demonstrated a differential influence of Wnt signaling in regulating the growth and vascular specification of CPCs during mouse cardiogenesis and cardiac differentiation of hPSCs. By combining recombinase-based fate mapping and cardiac-specific perturbations of Wnt signaling with *in vitro* modulation of Wnt signaling in hPSC differentiation cultures, we linked inhibition of Wnt signaling with acquisition of vascular fate, and identified a novel mechanism of cardiac neovascularization that is mediated by paracrine modulation of Wnt signaling in CPCs (Fig. 8).

The cellular origins of the coronary vasculature and its developmental patterning are relatively unexplored areas that have important implications for treatment of cardiovascular disease. Although endocardium has previously been thought to provide negligible contribution to myocardial vessels (Ishii et al., 2009), numerous groups have since demonstrated that endocardium undergoes angiogenic sprouting to generate endothelial networks within the coronary vascular tree (Del Monte and Harvey, 2012; Wu et al., 2012; Zhang and Zhou, 2013). Indeed, endocardial ECs in the fetal human heart have been shown to exhibit tip cell behavior, with endothelial networks in the myocardium sprouting from endocardial progenitors (Rusu et al., 2015). Therefore, increased expression of transcripts related to Notch signaling and tip cell phenotype in hPSC-derived NkxECs (Fig. 3B) might derive from an angiogenic impetus that is native to endocardium. Notch is known to provide a unique regulatory input in endothelial tip cells during neoangiogenesis (Benedito et al., 2009), and precise temporal regulation of Notch signaling between endocardium and myocardium coordinates ventricular patterning with coronary vessel development (D'Amato et al., 2016). Application of hPSC-derived endocardium in cell-based *in vitro* models of endocardial/myocardial crosstalk might provide a means of dissecting mechanisms of cardiac neovascularization and repair.

Although few studies have examined the effect of Wnt signal modulation on vascular specification of CPCs, pan-EC recombination strategies using Tie2-Cre and Cdh5-Cre mice have examined the effect of EC-specific gain/loss of β-catenin function (Corada et al., 2010). Although these mutations are not specifically targeted to heart vasculature, Corada et al. (2010) attributed the lethal phenotype of β-catenin^GOF^ embryos to increased Notch signal activation, which has been shown to reduce expression of Flk1 (Vegfr2 or Kdr) (Suchting et al., 2007; Williams et al., 2006). Given the essential role of Notch in tip cells during neoangiogenesis (Benedito et al., 2009), increased expression of tip cell genes in NkxECs (Fig. 3B) might reflect a role for Notch during vascular specification of CP/CMs. Indeed, Flk1 is an essential mediator of EC identity and absolutely required for acquisition of EC fate (Shalaby et al., 1997), so Wnt crosstalk with Flk1 via Notch could indirectly influence vascular specification of CP/CMs. Lower levels of FLK1 in CP/CMs cultured with CHIR99021 (Fig. 6E) and increased FLK1 in response to WNT5A or WNT11 (Fig. 6F) are consistent with an influence of Wnt signal activation on FLK1 in hPSC-derived CP/CMs. However, further experiments will be required to precisely define the mechanistic link between Wnt, Notch and FLK1 at the interface of CPC and EC identity.

Multiple studies have investigated the effect of β-catenin gain/loss of function during murine cardiogenesis (Klaus et al., 2007; Kwon et al., 2007). These studies revealed the multiphasic nature of Wnt, with loss of β-catenin later in cardiogenesis resulting in diminished right ventricles (derived from second heart field), whereas left ventricles (derived from first heart field) are of normal size. β-catenin^LOF^ embryos described in the present study (Fig. 5D-F) recapitulated previously reported anatomical aberrations, but also displayed a shift in the NkxEC:nEC ratio toward NkxECs specifically in the right ventricle. Distinct EC subgroups within the embryonic heart are difficult to parse, as they are believed to arise from multiple populations, including pharyngeal mesoderm and progenitors within the first and second heart fields (Harris and Black, 2010; Vincent and Buckingham, 2010). Our measurements of EC ratio during mouse cardiogenesis identified a substantial contribution of both NkxECs and nECs to endocardium; however, NkxECs were relatively enriched in the left ventricle (derived primarily from first heart field). Combined with the fact that conditional ablation of β-catenin significantly increased the proportion of NkxECs only in the left ventricle, these data suggest that Nkx2.5-expressing progenitors make a more substantial contribution to left ventricular endocardium. A shortcoming of this study is the lack of correlative Cre-based strains that enable manipulation of the nEC compartment. However, the finding that Wnt5a gain of function increased the proportion of NkxECs in both ventricles suggests that Wnt inhibition via secreted non-canonical ligands amplifies specification of NkxECs and not nECs from their respective progenitor populations. This could stem from a differential response to Wnt signaling among EC-competent progenitors or could be due to the fact that nECs may originate from progenitors that have undergone vascular specification outside the region of Wnt5a expression.

Previous studies have demonstrated a requirement for Wnt5a and Wnt11 in cardiac development (Bisson et al., 2015; Cohen et al., 2012), and both ligands exert an inhibitory influence on Wnt signaling in cells expressing the receptor Ror2 (van Amerongen et al., 2012). Wnt5a expression has also been linked to β-catenin-mediated Wnt activation in epicardium: knockout of *Wt1* results in reduced expression of Wnt5a as well as β-catenin targets (von Gise et al., 2011); and Wnt activation is accompanied by upregulation of Wnt5a in transient fibrotic tissue that ultimately gives rise to regenerated cardiomyocytes following cryoinjury of neonatal heart (Mizutani et al., 2016). In the present study, inhibitory effectors of canonical and non-canonical Wnt signaling were significantly enriched in CP/CMs (Fig. 2B,C), and exposure to exogenous WNT5A and WNT11 in isolated CP/CMs, or ectopic Wnt5a in embryonic mouse heart, enriched for NkxECs (Fig. 5). Although a vascular phenotype in embryonic hearts of *Wnt5a* and/or *Wnt11* mutant mice has not been addressed, the data above suggest that non-canonical Wnt plays a role in not only morphogenesis and proliferation, but also vascular specification within embryonic heart.

Although there is no precedent for Wnt activation regulating FLK1 expression in CPCs, recent work has expanded the scope of Wnt signaling during cardiogenesis, elucidating its function in balancing the differentiation and proliferation of epicardial progenitor-like cells from hPSCs (Iyer et al., 2016; Witty et al., 2014). These studies demonstrate a transient growth phase following specification of CPCs, during which cells progress through proepicardial intermediates to self-renewing epicardial cells that generate smooth muscle cell (SMC) and fibroblast derivatives. Although differentiation of hPSC-derived epicardium to EC fate is negligible and numerous studies suggest that epicardium in development mostly contributes to cardiomyocytes, SMCs and fibroblasts (Cai et al., 2008; Zhou et al., 2008a), fate-mapping experiments have clearly demonstrated that the proepicardial organ derives from Nkx2.5-expressing progenitors (Zhou et al., 2008b) and makes a substantial contribution to the coronary vasculature (Katz et al., 2012). Indeed, a previous study of epicardial-specific null mutation of β-catenin demonstrated a failure of epicardial cells to colonize and expand within the subepicardial space, ultimately resulting in a lack of subepicardial vascularization (Zamora et al., 2007). Given the prominent contribution that epicardium makes to the cellular constitution of the cardiac wound healing response, interrogation of pathways that modulate the differentiation potential of epicardial progenitors might provide a means of improving the recovery of functional cardiac tissue following ischemic injury.

Many groups have linked inhibition of Wnt signaling to improved wound healing following MI. Transgenic mice overexpressing the Wnt inhibitor SFRP1 showed reduced scar formation and improved cardiac function following MI with significantly higher capillary density observed in transgenic hearts (Barandon et al., 2004, 2003). Direct administration of recombinant SFRP2 (He et al., 2010) or SFRP4 (Matsushima et al., 2010) into the heart post-MI reduced fibrosis and improved cardiac function in a dose-dependent manner, resulting in better infarct healing. This benefit is recapitulated in cell-autonomous models of Wnt loss of function: conditional depletion of β-catenin in adult hearts following MI results in mobilization of tissue-resident CPCs, increased 4-week survival and improved left ventricular function (Zelarayan et al., 2008). Pharmacological inhibition of Wnt also provides a benefit during cardiac wound healing: Pyrvinium, an FDA-approved drug that has been shown to exert an inhibitory influence on Wnt, improved cardiac function at 1 month post-MI (Saraswati et al., 2010), and a peptide fragment that acts as a competitive inhibitor of Wnt signal activation (UM206) reduced infarct size and improved cardiac function and survival following MI, with a significant increase in neovascularization of the infarct zone (Laeremans et al., 2011). Finally, two recent studies have employed pharmacological inhibitors of porcupine, a protein required to enable exit of Wnt ligands from the endoplasmic reticulum, to demonstrate a beneficial effect of global Wnt inhibition on cardiac remodeling and function post-MI (Bastakoty et al., 2016; Moon et al., 2017). Although Wnt signaling is likely to play a role in numerous pathological responses to cardiac wound healing, including inflammation, proliferation and remodeling, these results might reflect a capacity for Wnt signal inhibition to enforce pro-angiogenic differentiation among adult CPCs.

## MATERIALS AND METHODS

### Human pluripotent stem cell (hPSC) culture

Experiments were performed using the Mel-1 *NKX2.5*^eGFP/w^ hPSC line (Elliott et al., 2011), which was provided by David Elliot, Andrew Elefanty and Edouard Stanley. Permission for use of this cell line was granted by the Cornell-Rockefeller-Sloan Kettering Institute ESCRO committee. hESC culture medium consisted of Advanced DMEM/F12 (Gibco) with 20% Knockout Serum Replacement (Invitrogen), L-glutamine (2 mM, Invitrogen), Pen/Strep (Invitrogen), β-mercaptoethanol (550 nM, Gibco), and 4 ng/ml FGF2 (Invitrogen). hESCs were maintained on Matrigel (Corning) using MEF-conditioned hPSC medium (GlobalStem) and handled as previously described (James et al., 2006). All cell lines were confirmed to be mycoplasma free every ten passages.

### Lentiviral transduction

Lentiviruses were generated by transfecting 15 µg lentiviral vector, 3 µg pENV/VSV-G, 5 µg pRRE, and 2.5 µg pRSV-REV in HEK 293T cells (passage 8-10; subconfluent, 100 mm dish) by the calcium precipitation method. Supernatants were collected 40 and 64 h after transfection and concentrated by Lenti-X concentrator (Clontech, 31232). Using *NKX2.5*^eGFP/w^ hPSCs, clonal lines containing the VPr^mOrange^ EC-specific transgene were obtained as previously described (James et al., 2011).

### hPSC differentiation

One day before plating hPSCs to begin differentiation, MEF-conditioned medium was replaced with hPSC culture medium without FGF2 and supplemented with 2 ng/ml BMP4. The next day (day 0 of differentiation), hPSCs were plated directly onto gelatin in hPSC culture medium (without FGF2, plus 20 ng/ml BMP4, 40 ng/ml activin A, 2.5 µM CHIR99021) and not disturbed for 48 h. On day 2, cells were stimulated with 40 ng/ml BMP4 and 2 ng/ml FGF2. Differentiation cultures containing VEGFA at 10 ng/ml beginning from day 2 to point of harvest. In isolated CP/CM cultures, VEGFA was not included during bulk hPSC differentiation. CHIR99021, Endo-IWR1 and XAV-939 were used at 2.5 µM unless otherwise indicated. Recombinant cytokines (Wnt5a, Wnt11; R&D Systems) were used at 100 ng/ml.

### Lentivirus-based reporter assay

FACS-sorted CP/CMs were plated into individual wells of a 48-well flat-bottom plate in hPSC base medium with 10 ng/ml VEGFA and other recombinant cytokines as indicated. Individual wells were transduced overnight with *TCF/Lef* lentiviral particles expressing firefly luciferase or the respective positive and negative control lentiviral particles (Cignal Lenti Reporter Kit, SABiosciences) at a multiplicity of infection of 25 according to the manufacturer's instructions. After 18 h, medium was removed and CP/CMs were incubated in designated conditions for 3 days further, changing the medium after 2 days. Four days following isolation, luciferase activity was measured on a SpectraMax M5 microplate reader (Molecular Devices) using the Steady-Glo Luciferase Assay System (Promega) according to the manufacturer's instructions.

### RNA-seq library construction, sequencing and analysis

Total RNA was prepared from FACS-sorted cells and quality was checked on an Agilent Technologies 2100 Bioanalyzer. 1 µg high-quality total RNA was used as input to convert mRNA into a library of template molecules for subsequent cluster generation and sequencing using the reagents provided in the Illumina TruSeq RNA Sample Preparation Kit. Following purification of the poly(A)-containing mRNA using poly(T) oligo-attached magnetic beads, the mRNA was fragmented into small pieces using divalent cations under elevated temperature. The cleaved RNA fragments were copied into first-strand cDNA using reverse transcriptase and random primers, followed by second-strand cDNA synthesis using DNA polymerase I and RNase H. These cDNA fragments then went through an end-repair process, the addition of a single ‘A’ base, and then ligation of adapters. The products were purified and enriched by PCR to create the final cDNA library. After quantifying and checking the size and purity of the product, multiplexed DNA libraries were normalized to 10 nM and then two sample libraries were pooled together in equal volumes. 7 pM each pooled DNA library template was amplified on an Illumina cBot instrument, involving immobilization and 3′ extension, bridge amplification, linearization and hybridization, then sequenced on one lane of the Illumina HiSeq2000 sequencer using the pair-end module and generating 2×58 bp reads. The samples HUVEC, CP/CM, NkxEC and nEC were aligned to the human GRCh38 reference assembly (NCBI) using STAR aligner (Dobin et al., 2013) and subsequently genes were counted in htseq (Harada et al., 1999). The raw counts mapping to GRCh38 were subsequently analyzed in R package edgeR and differential expression analysis was performed. After selecting the most variant 910 genes by expression, multidimensional scaling (MDS) plots were prepared among the sample groups, and showed clear differentiation between the four tissue types (Fig. 3A).

### FACS

Flow cytometry analysis and sorting was performed on a FACSJazz (BD Biosciences). Configuration of the FACSJazz was as follows: 405 nm laser with 450/50 band pass (BP) (DAPI) and 520/35 BP (BV 510) filters; 488 nm laser with 530/40 BP (GFP) and 585/29 BP (PE) filters; and 640 nm laser with 660/20 BP (APC) and 750 LP (APC-Cy7) filters. Antibodies used were specific for human CD31-PE (MEC 13.3, BD Biosciences), human CD43-APC (10G7, Biolegend), mouse CD31-PE (390, eBioscience) and mouse CD45-APC (30-F11, eBioscience); antibodies were used at the concentration recommended by the manufacturer. Data were analyzed using FACS Diva software (BD Biosciences). Clonal sorting was via direct deposition of five cells per well of a 96-well plate using the FACSJazz Automated Cell Deposition Unit (ACDU).

### Immunofluorescence

Cells were stained as previously described (James et al., 2010). Samples were fixed in 4% paraformaldehyde, permeabilized in PBST (0.1% Tween 20) and blocked in 5% donkey serum for 45 min. Samples were incubated for 2 h in blocking solution containing primary antibodies that were directly conjugated to Alexa 488, 568 or 647. Antibodies used were specific for CD31 (MEC 13.3, BD Biosciences), Nkx2.5 (8792, Cell Signaling), cardiac troponin (MAB6887, R&D Systems) and smooth muscle actin (MAB1420, R&D Systems). Imaging was performed using a Zeiss LSM 710 confocal microscope.

### Confocal microscopy and live imaging

Microscopy was performed using a 710 META confocal microscope (Zeiss) with the following laser lines: 405, 458, 488, 514, 561 and 633 nm. Filter settings for fluorescent proteins and fluorophores were: 450/20 BP for DAPI; 530/20 BP for GFP and Alexa 488; 560/20 BP for mOrange; 630/30 BP for tdTomato; and 670/20 BP for Alexa 647. For live imaging, *NKX2.5*^eGFP^/VPr^mOrange^ hPSCs were cultured at 37°C and 5% CO_2_ in a stage-mounted incubation chamber (Tokai Hit), with image capture at variable intervals. Colocalization was performed using Zen colocalization software (Zeiss) and the pixel area of single- and double-positive cells quantified.

### Mouse strains

All procedures were approved by the Institutional Animal Care and Use Ethics Committee of Weill Cornell Medical College. Breeding was performed using animals between 12 weeks and 1 year of age. The mouse strains that were intercrossed to generate the animals in this study were: *Flk1-GFP* (Jackson Labs, 017006), *Rosa*^tdTom^ (Jackson Labs, 007905), TCF:LEF^H2B-GFP^ (Jackson Labs, 013752), *Cdh5*^Cre^ (Jackson Labs, 017968), β-catenin^flox/flox^ (Jackson Labs, 004152), *Igs1^tm6(CAG-Bgeo,-Wnt5a)Nat^* (*Wnt5a*^GOF^; Jackson Labs, 018141), *Wnt5a*^flox/flox^ mice (Jackson Labs, 026626), *Nkx2.5*^Cre^ [provided by Richard Harvey (Stanley et al., 2002)] and β-catenin^flEx3/flEx3^ [provided by Makoto Taketo (Harada et al., 1999)]. For quantification of NkxEC versus nEC number, 10 µm sections were collected for the entire span of the heart and colocalization masking distinguished EC subtypes.

### Statistical analysis

Experiments were repeated at least three times, with the technician performing quantitative analysis blinded to the experimental conditions. We included all tested animals for quantification to analyze the statistical difference. A representative image from experimental groups is presented in corresponding figures. No statistical method was used to predetermine sample size. Differences between groups containing more than one condition were assessed by one-way ANOVA, and to quantify statistical difference of each condition to control an unpaired two-tailed *t*-test was performed.

## Supplementary Material

Supplementary information

Supplementary information
