# Supplementary material for: Wnt inhibition promotes vascular specification of embryonic cardiac progenitors
Source: Development. 2018 Jan 1;145(1):dev159905. doi: 10.1242/dev.159905 (PMC5825863; doi:10.1242/dev.159905)
Supplement: Supplementary information [file develop-145-159905-s1.pdf]

Supplementary Movies

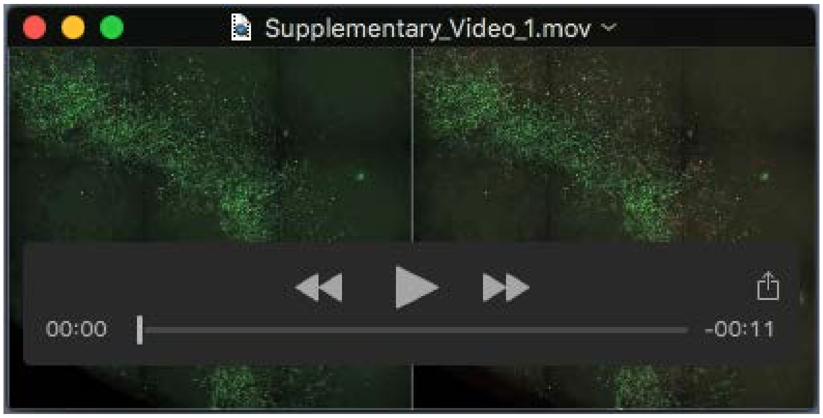

Movie 1. Wide field time-lapse view of dual reporting hPSCs during cardiovascular differentiation. Following 8 days of differentiation, a tiled view of an hPSC-derived colony was captured at 6-minute intervals over 48 hours. Nkx2.5^{GFP+} cells are shown in green, VPr$^{mOrange+}$ cells are shown in red. A higher magnification video of the region indicated by the stroke box is shown after the first sequence.

Development • Supplementary information

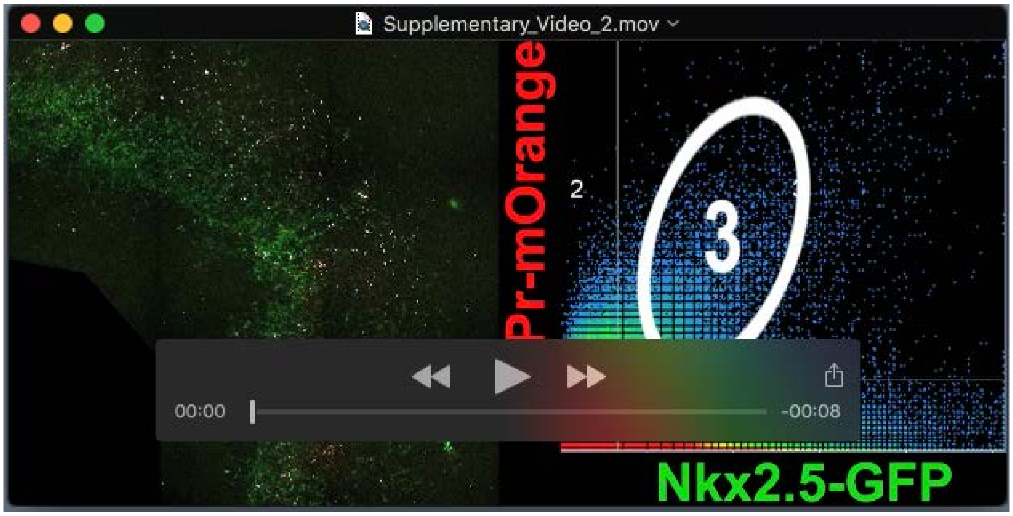

Movie 2. Dual-reporting hPSCs enable identification of cells during transition from CPC to NkxEC fate. Beginning at 8 days of differentiation, cells expressing both Nkx2.5^{GFP} and VPr^{mOrange} were identified by co-localization masking (shown in white). The plot on the right identifies signal intensity for Nkx2.5^{GFP} and VPr^{mOrange} pixels on the x-axis (1) and y-axis (2), respectively. Pixels expressing both transgenes are designated in field 3.

Development • Supplementary information

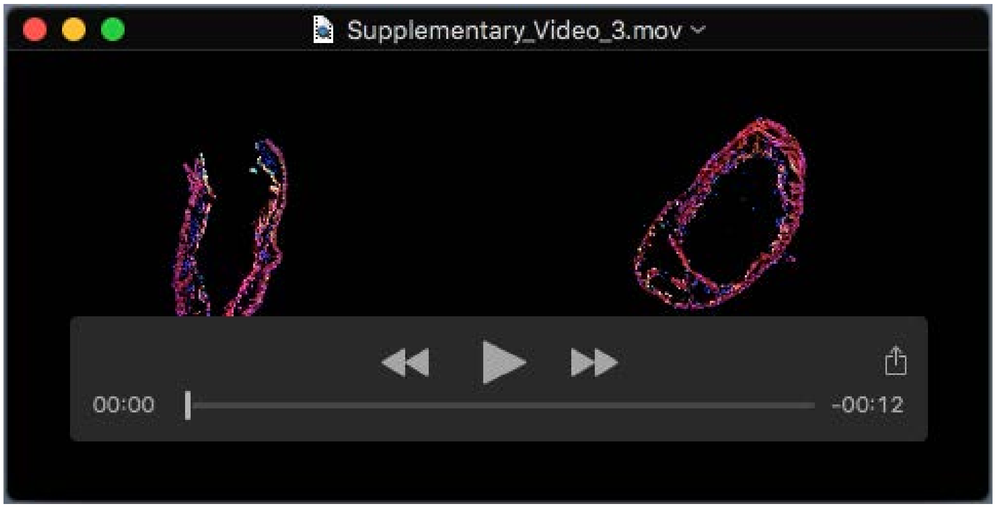

Movie 3. β-catenin null embryonic hearts display reduced right ventricular size and increased percentage of NkxECs. Embryonic hearts from heterozygous (left) and homozygous (right) β-catenin null mutants were serially sectioned and imaged by confocal microscopy. Nuclei (blue), $VEGFR2^{GFP+}$ ECs (green) and $Nkx2.5^{Cre}$:$tdTomato^{+}$ cells (red) are shown.

Development • Supplementary information

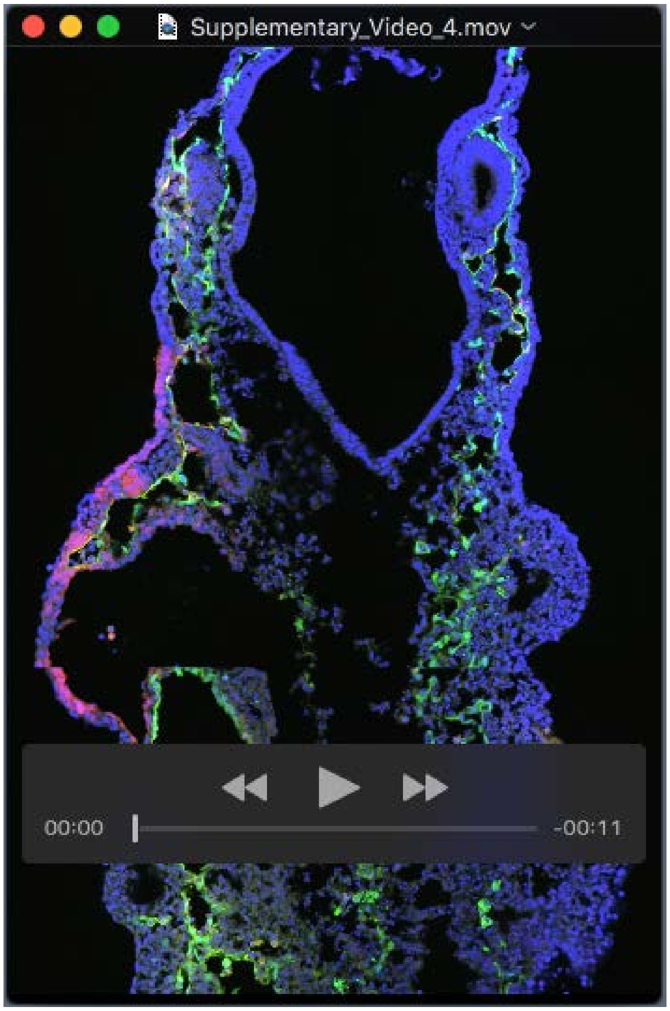

Movie 4. β-catenin gain of function embryos at e11.5 display aberrant cardiac development and reduced NkxECs. β-catenin gain of function mutant embryo was serially sectioned and imaged by confocal microscopy. Nuclei (blue), VEGFR2^{GFP+} ECs (green) and Nkx2.5^{Cre}:tdTomato^{+} cells (red) are shown.

Development • Supplementary information

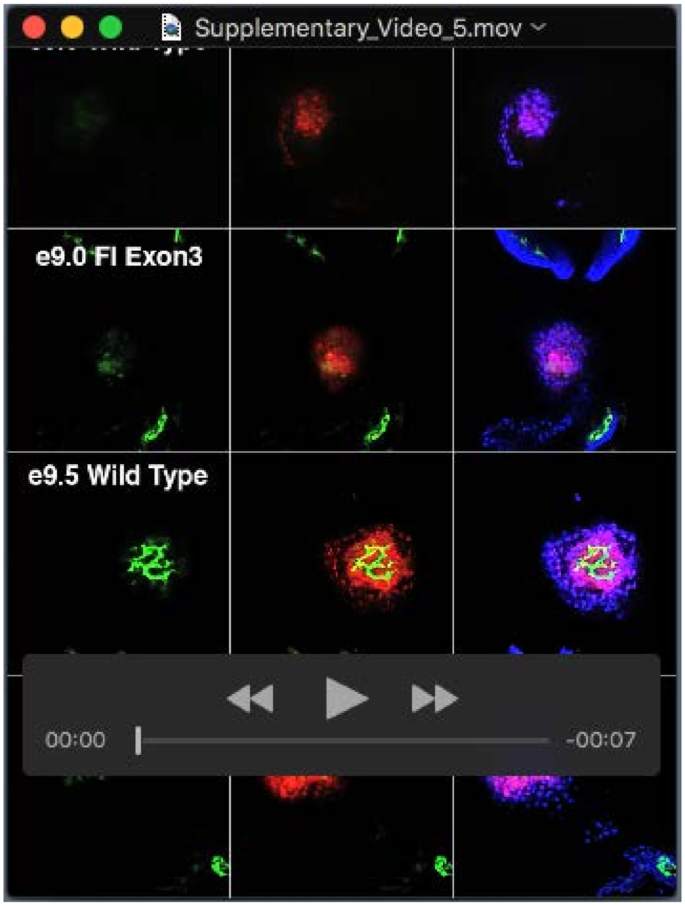

Movie 5. β-catenin gain of function embryos at e9.0 and e9.5 show reduced vascular density. Confocal z-stacks were captured for WT and β-catenin gain-of-function mutants at e9.0 (upper) and e9.5 (lower).

Development • Supplementary information

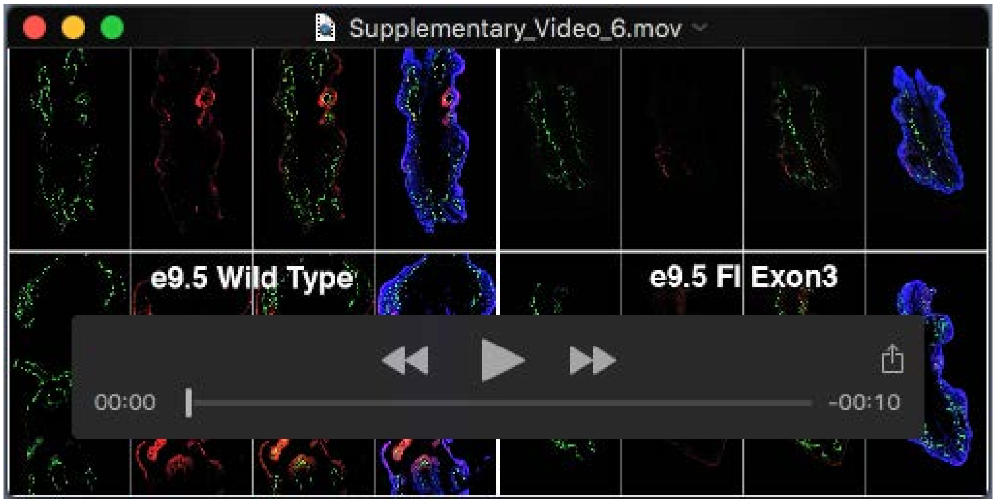

Movie 6. β-catenin gain of function embryos at e9.0 and e9.5 show reduced vascular density. Embryos from wild type and heterozygous β-catenin gain of function embryos were serially sectioned and imaged by confocal microscopy. Nuclei (blue), VEGFR2^{GFP+} ECs (green) and Nkx2.5^{Cre}:tdTomato^{+} cells (red) are shown.

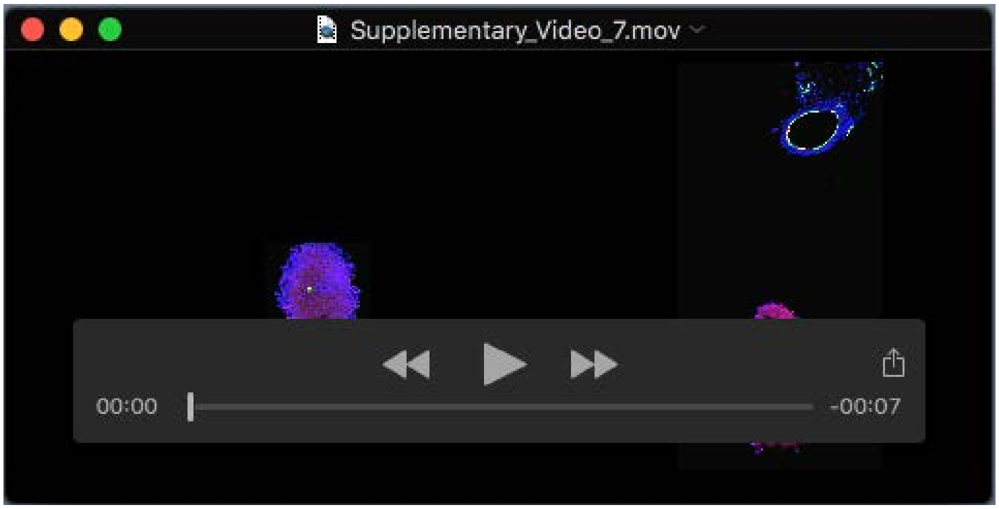

Movie 7. Wnt5a^{GOF} hearts display morphological defects, reduced ventricular trabeculation and increased percentage of NkxECs. Embryonic hearts from control (left) and Wnt5a^{GOF} (right) hearts were isolated at e12.5, serially sectioned and imaged by confocal microscopy. Nuclei (blue), VEGFR2^{GFP+} ECs (green) and Nkx2.5^{Cre}:tdTomato^{+} cells (red) are shown.

Development • Supplementary information
